# *Staphylococcus aureus* vWF-binding protein triggers a strong interaction between clumping factor A and host vWF

**DOI:** 10.1038/s42003-021-01986-6

**Published:** 2021-04-12

**Authors:** Albertus Viljoen, Felipe Viela, Marion Mathelié-Guinlet, Dominique Missiakas, Giampiero Pietrocola, Pietro Speziale, Yves F. Dufrêne

**Affiliations:** 1 Louvain Institute of Biomolecular Science and Technology, UCLouvain, Louvain-la-Neuve, Belgium; 2 Department of Microbiology, The University of Chicago, Chicago, IL USA; 3 Howard Taylor Ricketts Laboratory, Argonne National Laboratory, Lemont, IL USA; 4 Department of Molecular Medicine, Unit of Biochemistry, University of Pavia, Pavia, Italy; 5 Walloon Excellence in Life Sciences and Biotechnology (WELBIO), Wavre, Belgium

**Keywords:** Single-molecule biophysics, Single-molecule biophysics, Bacteriology, Pathogens, Bacterial pathogenesis

## Abstract

The *Staphylococcus aureus* cell wall-anchored adhesin ClfA binds to the very large blood circulating protein, von Willebrand factor (vWF) via vWF-binding protein (vWbp), a secreted protein that does not bind the cell wall covalently. Here we perform force spectroscopy studies on living bacteria to unravel the molecular mechanism of this interaction. We discover that the presence of all three binding partners leads to very high binding forces (2000 pN), largely outperforming other known ternary complexes involving adhesins. Strikingly, our experiments indicate that a direct interaction involving features of the dock, lock and latch mechanism must occur between ClfA and vWF to sustain the extreme tensile strength of the ternary complex. Our results support a previously undescribed mechanism whereby vWbp activates a direct, ultra-strong interaction between ClfA and vWF. This intriguing interaction represents a potential target for therapeutic interventions, including synthetic peptides inhibiting the ultra-strong interactions between ClfA and its ligands.

## Abstract (web summary)

Through force spectroscopy studies on living bacteria, Viljoen et al. characterise the binding of *S. aureus* to host von Willebrand factor (vWF). They propose that *S. aureus* vWF-binding protein triggers an ultra-strong interaction between the adhesin clumping factor A and vWF.

## Introduction

**Fig. 1:**
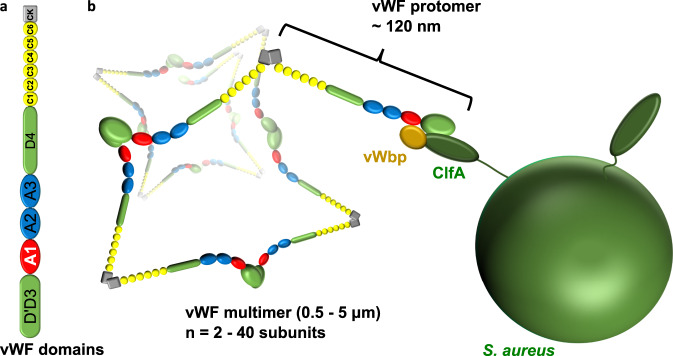
*Staphylococcus aureus* adhesion to von Willebrand factor (vWF). **a** The modular architecture of mature vWF. vWF-binding protein (vWbp) binds to the A1 domain. **b** Cartoon depicting *S. aureus* ClfA and vWbp in a hypothetical complex with vWF under the shear stress of arterial blood flow. Tangled and coiled globular vWF is stretched out by the arterial flow. vWbp mediates ClfA-dependent binding of *S. aureus* to vWF via its A1 domain.

Staphylococci produce a range of adhesins that promote high-affinity interactions with human circulatory proteins^1–3^. In this manner, these pathogens adhere to blood vessel endothelia despite the shear stress from flowing blood and can cause life-threatening vascular infections. A prototypical example is the binding of *Staphylococcus aureus* adhesins to the blood circulatory protein von Willebrand factor (vWF)^4–7^. vWF is a multimeric glycoprotein made of identical monomers with a modular architecture. From N- to C-terminus, each mature monomer comprises domains D′D3, A1, A2, A3, D4, B1-3, C1-8, and CK; domains A1 and A3 bind platelet Glycoprotein Ibα [GpIb] and collagen, respectively, and domain C4 contains an RGD repeat that binds to the integrin α_IIb_β_3_^8,9^ (Fig. 1a). vWF multimerization is triggered by the sequential formation of disulfide bonds between the C-terminal CK domains of two protomers leading to tail-to-tail homodimerization followed by disulfide linkage between N-terminal D3 domains of adjacent dimers^10,11^. This self-assembly process results in the formation of right-handed helical tubules that are stocked in the Weibel-Palade bodies of endothelial cells, from which they are ultimately secreted into the circulation^10,12^. As a result of this extensive multimerization, plasma vWF may contain up to 40 subunits and be up to several microns long when secreted at basal levels^13^. Further, newly secreted vWF multimers adopt a relatively compact globular state of tangled coils^13^, which under high shear flow (e.g., in the arteries) extend out, exposing sites for platelet recruitment and for extracellular matrix protein binding (e.g., collagen)^14–16^.

Two *S. aureus* proteins have been proposed to interact directly with vWF, cell-wall-bound Staphylococcal protein A (SpA)^17^ and secreted vWF-binding protein (vWbp)^5,18^. SpA binds to both the vWF-A1 and D′D3 domains via four of its five Ig-binding N-terminal repeat domains^19^. We have demonstrated that the vWF-SpA interaction is mechanically regulated and can resist very high-force loads (~2 nN)^20,21^. vWbp, like SpA, binds the vWF-A1 domain via a 26-amino acid sequence in its C-terminus^18^. However, unlike SpA, which harbors a cell-wall attachment sequence at its carboxyl terminus^22^, secreted vWbp does not bind covalently to the cell wall^18^. Cell-wall-associated binding partners of vWbp include wall teichoic acids^4^ and the sortase-dependent cell-wall-anchored adhesin, clumping factor A (CflA)^6^. In vitro as well as in vivo perfusion experiments demonstrated that vWbp/ClfA-dependent adhesion of *S. aureus* to vWF is augmented by shear stress^5,6^. This could be because vWF extends under shear, revealing vWbp-binding sites that promote increased bacterial binding. Alternatively, vWF, vWbp, and ClfA could form a ternary complex whose stability is mechanically activated by shear stress (Fig. 1b). ClfA is a member of the microbial surface components recognizing adhesive matrix molecules (MSCRAMMs) family of proteins that characteristically contains two adjacent IgG-like folded N-terminal subdomains^2^. Some MSCRAMM members bind with high affinity to the blood plasma protein fibrinogen (Fg) via a “dock”, “lock”, and “latch” (DLL) mechanism, which was first unraveled from crystal structures of the *S. epidermidis* ClfA-orthologue, SdrG in complex with an N-terminal peptide of the Fg β-chain^23,24^. In the case of SdrG, the DLL mechanism can be described in three steps: (i) docking of the Fg β-chain N-terminal peptide in a pocket located between the N2 and N3 domains of SdrG, (ii) folding of a C-terminal extension of the N3 domain over the docked ligand, and (iii) latching of a part of this extension onto the N2 domain where it complements a β-sheet^23,24^. Recently, single-molecule experiments and molecular dynamics simulations demonstrated that the resulting snug confinement of the Fg peptide creates a shear geometry such that multiple hydrogen bonds must be broken in parallel to pull Fg out of its binding pocket thus explaining the remarkable stability of the complex^25,26^. ClfA binds a C-terminal sequence of the Fg γ-chain in a similar manner via a variation of the DLL mechanism^27,28^.

Adhesion of *S. aureus* to vWF under shear stress has been unraveled using bacterial mutants that lacked functional vWbp or ClfA^5,6^. Clearly, an interaction between these three factors is critically relevant for pathogenesis. However, these findings left unresolved the molecular basis for vWF’s interaction with vWbp and ClfA. Here, we propose that vWF-vWbp-ClfA form a ternary complex. We use atomic force microscopy (AFM) and single-molecule experiments with live cells to examine the specific range of forces withstood by this complex; we also measure the stability of this complex under mechanical stress. Last, we evaluate models whereby vWbp may either form a bridge between vWF and ClfA or trigger a direct interaction between the two proteins.

## Results

### The vWF-vWbp-ClfA ternary complex ruptures in the nanonewton range

**Fig. 2:**
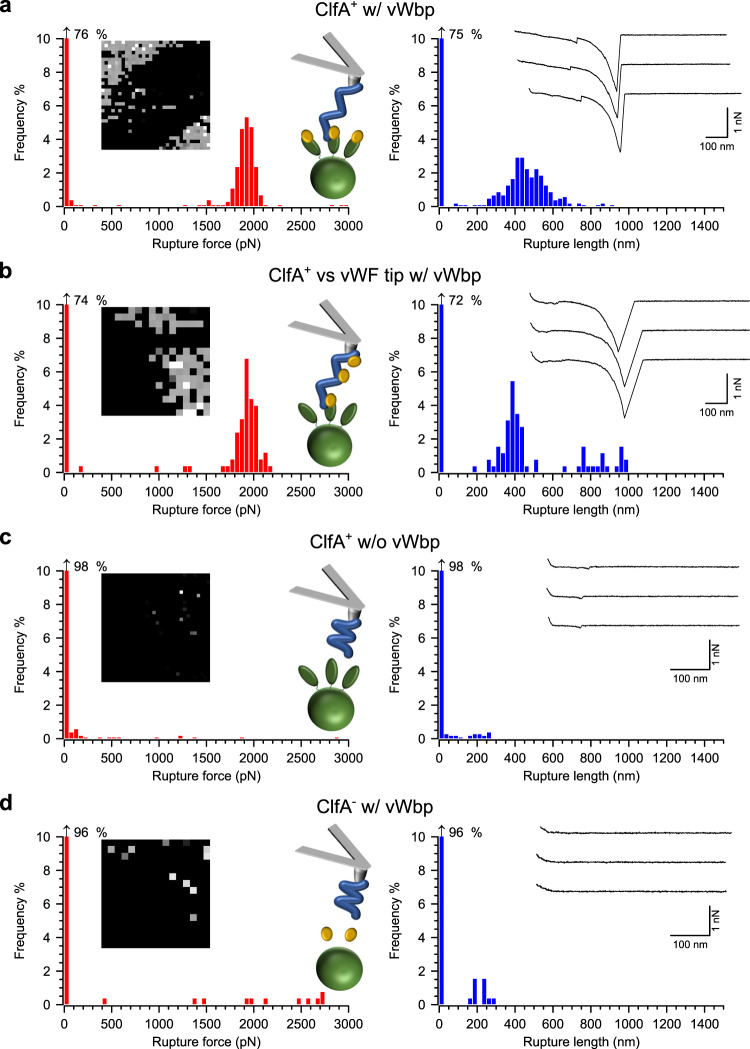
vWbp augments ClfA-dependent binding of *S. aureus* to vWF and the ternary complex is very strong. **a** *S. aureus* cells expressing ClfA at high levels and treated with recombinant vWbp probed with vWF-modified AFM tips. Data for a representative cell are shown. For more cells, see Supplementary Fig. 1. On the left are histograms of rupture forces with insets showing the respective adhesion maps (500 × 500 nm, 32 × 32 or 16 × 16 pixels, gray scale = 0–3 nN, each dot represents a binding event) and a cartoon in the top graph illustrates the experimental setup. Green ovals represent ClfA, golden spheres vWbp, and blue lines vWF. On the right are shown histograms of the rupture lengths with insets showing three representative retraction profiles. **b** Data for ClfA^+^ *S. aureus* cells (not treated with vWbp) probed with vWF-functionalized tips treated with vWbp. **c** Data for vWbp-untreated ClfA^+^ cells probed with vWF-modified tips. **d** Data for vWbp-treated ClfA^−^ cells probed with vWF-modified tips.

What is the binding strength of the vWF-vWbp-ClfA ternary complex? Single *S. aureus* cells were incubated with purified vWbp and probed with AFM tips modified with vWF. To avoid SpA-dependent vWF-binding, which occurs at a frequency of ~15% when using strain *S. aureus* Newman^20^, we used a variant of *S. aureus* SH1000, a laboratory strain that produces reduced amounts of SpA^29^, and over-produces ClfA from a multicopy plasmid (for detailed strain descriptions, see “Methods”)^28,30^. When treated for 15 min with purified vWbp, cells of this new strain referred to as ClfA^+^ bound vWF with a very high frequency (40 ± 17%, *n* = 14 cells, Fig. 2a, Supplementary Table 1); such a frequency is in line with previous measurements for the ClfA-Fg interaction using the same strain^28^. We routinely observed very high binding frequencies despite limiting the contact time between the vWF-modified tips and the *S. aureus* cells (see “Methods”). This is in contrast with previous work exploring the SpA-vWF interaction where it was necessary to increase the contact time to observe binding^20^. Thus, our results suggest that the vWF-vWbp-ClfA complex forms more rapidly than the vWF-SpA complex. This is in agreement with the report by Claes et al. showing that under shear a *spa* mutant did not show reduced binding to vWF, while *clfA* or *vwb* (encoding ClfA and vWbp, respectively) mutants clearly bound vWF less than the isogenic wild type strain^6^. Interestingly, ClfA appeared to cluster in domains on the bacterial cell surface (Fig. 2a) reminiscent of other *S. aureus* MSCRAMMs^31^. This clustering may play a role in enhancing bacterial adhesion. As shown in Fig. 2a, the magnitudes of forces were narrowly distributed within a single Gaussian peak centering at 1997 ± 359 pN (mean ± standard deviation [s.d.], *n* = 2021 adhesive curves from 14 cells, Supplementary Table 1, Supplementary Fig. 1). The unimodality of the force distributions as well as the observation of mainly single force peaks in our force-distance curves (e.g., Fig. 2a, right panel, force curve insets) confirm that we mainly probed interactions involving single complexes. Measurements of the rupture lengths, which approximate the length of the extended complex, showed a broad distribution ranging from ~250 to ~800 nm (*n* = 2021 adhesive curves from 14 cells, Supplementary Table 1). Considering that the average length of an elongated vWF protomer is ~120 nm^13^, cell-wall-bound ClfA ~160 nm^27^, and fully unfolded vWbp (based on its polypeptide sequence) ~170 nm, it appears that we mainly stretched short segments of vWF oligomers. In addition, the broad range of rupture lengths observed is a reflection of the randomness of the free-to-be-extended stretches of vWF that we achieved during tip functionalization (see “Methods”). Of note, the inverted configuration, in which vWF was incubated with vWbp rather than ClfA, resulted in similar adhesion features. Preincubation of vWF-modified tips with vWbp prior to using them to probe vWbp-untreated ClfA^+^ cells led to high binding frequencies (31 ± 21%, *n* = 6 cells, Fig. 2b and Supplementary Table 2) and high binding forces (1889 ± 83 pN and 543 ± 67 nm, *n* = 470 curves from six cells), in the same range as those of vWbp-treated ClfA^+^ cells probed with vWF tips.

To demonstrate the specificity of the ternary interaction we performed a series of control experiments. Probing vWbp-untreated ClfA^+^ cells with vWF-modified tips yielded very low binding probabilities at 4 ± 3% (*n* = 9 cells) (Fig. 2c, Supplementary Table 1). Similarly, treating cells lacking *clfA* (ClfA^−^ cells) with vWbp and then probing them with vWF-modified tips showed a very low frequency of binding at only 2 ± 2% (*n* = 10 cells) (Fig. 2d, Supplementary Table 1). These infrequent binding events may be attributed to residual vWF-SpA interactions^20^. These results strongly support the view that vWbp mediates the formation of an ultrastable ternary complex with vWF and ClfA.

### High mechanostability of the vWF-vWbp-ClfA complex

**Fig. 3:**
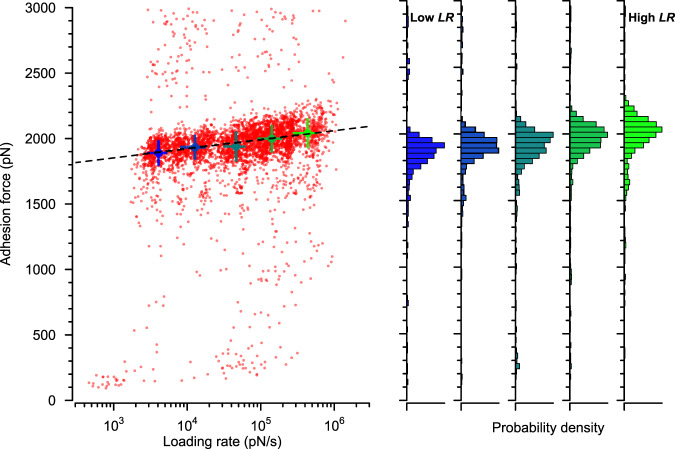
Force-loading rate dependence for the vWF-vWbp-ClfA ternary interaction follows Bell–Evans dynamics. Left: dynamic force spectroscopy plot of rupture force vs loading rate (*n* = 2992 curves from 3 cells, for individual data from 6 cells see Supplementary Fig. 2). The dotted line shows the extrapolated Bell–Evans fit through the most probable (mean) rupture forces and loading rates for five log-equispaced loading rate bins shown as solid circles. Error bars are the standard deviations. Right: corresponding histograms of the plot shown on the left.

What are the dynamics of the vWF-vWbp-ClfA interaction? To answer this question we performed dynamic force spectroscopy (DFS) experiments in which the retraction speed of the AFM tip was varied. ClfA^+^ cells were treated with vWbp, probed with vWF-modified AFM tips and DFS plots were recorded as shown in Fig. 3 (DFS plots of six individual cells are shown in Supplementary Fig. 2). The binding strength was found to increase with tensile load, as predicted by the Bell–Evans theory^32^.

A number of staphylococcal interactions show a bimodal distribution of lower and higher rupture forces, and a shift toward the higher regime under increased mechanical load^33^. For example, the interaction between ClfA and Fg is characterized by a high tensile force-tolerating state (ruptures at ~1500 pN) that is enhanced by physical stress and is mediated via the DLL mechanism, while a second low tensile force-tolerating state (ruptures at ~100 pN) may rely on an alternative Fg-binding site not involving DLL^28,34^. Similarly, in the ClfA-Fg-*α*_V_*β*_3_ integrin ternary bridge, tensile stress induces extension of Fg to unveil cryptic integrin-binding sites that form additional interactions with the integrin, thereby strengthening the interaction^35^. However, for the vWF-vWbp-ClfA ternary interaction, we only observed unimodal force distributions, both at low and high loading rates (Fig. 2a, Supplementary Fig. 1, Fig. 3), similar to the binary SdrG-Fg β-chain interaction^25,36^. This suggests that the mechanism behind the strength of the vWF-vWbp-ClfA interaction differs from those of previously investigated ternary complexes.

### The mechanical stabilities of the vWF-vWbp and vWbp-ClfA binary complexes are poor

**Fig. 4:**
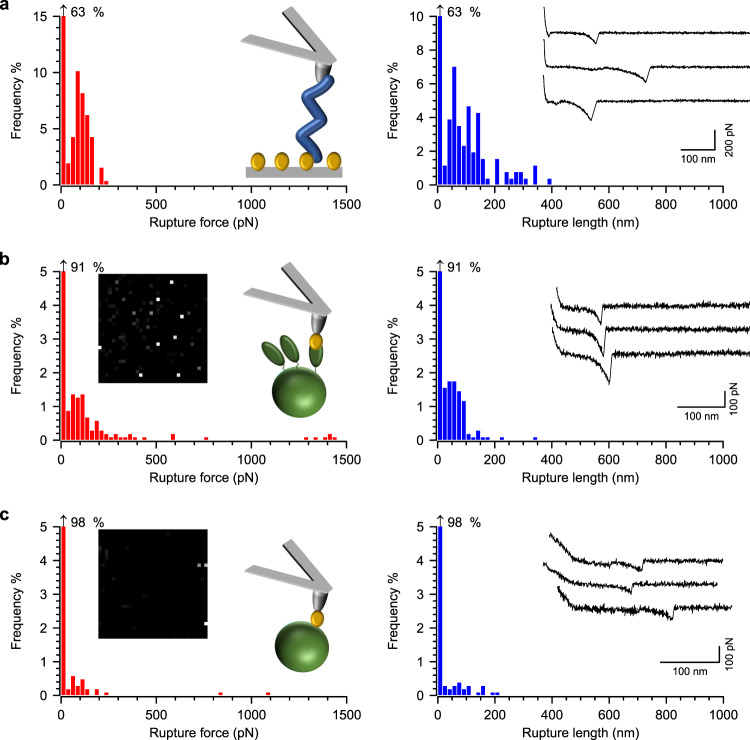
The vWbp-vWF and vWbp-ClfA binary interactions are much weaker than the ternary complex. **a** vWbp-modified surfaces probed with vWF-modified tips. Representative data—one surface-tip combination—are shown. Left panel: Histograms of rupture forces (recorded on a 10 µm × 10 µm area, 16 × 16 pixels) and a cartoon illustrating the experimental setup. Right panel. Histograms of the rupture lengths and insets of three representative curves. **b** *S. aureus* ClfA^+^ cells probed with vWbp-modified AFM tips. A pause after contact step of 500 ms (contact time) was included to enhance binding frequency. Data shown are for one cell-tip combination. Left panel: Histogram plot of rupture forces. Insets show the respective adhesion map (500 × 500 nm, 32 × 32, gray scale = 0–1.5 nN, each dot represents an adhesion event) and a cartoon to illustrate the experimental setup. **c** Data for a ClfA^−^ cell probed with a vWbp-modified tip, with 500 ms contact time.

In a ternary complex, we expect the weakest end to rupture first. We therefore asked whether the vWF-vWbp or vWF-ClfA binary complexes are as strong as the ternary complex. We first focused on the vWF-vWbp interaction. We modified AFM tips to expose vWF and gold supports to expose vWbp (Fig. 4a). This resulted in a relatively high frequency of binding (24 ± 18%, Supplementary Table 3), with rupture forces averaging at 136 ± 37 pN (mean ± s.d., *n* = 244 curves) and rupture lengths of 85 ± 25 nm. These forces are ~20 times lower than for the ternary complex. To study the vWbp-ClfA interaction, we probed ClfA^+^ cells with vWbp-modified tips (Fig. 4b), which resulted in low binding frequencies (3 ± 1%, *n* = 3 cells, Supplementary Table 4). Increasing the contact time before tip retraction from the minimum (~100 ms) to 500 ms led to a marked increase in binding (11 ± 5%, *n* = 11 cells and 4 tips), indicating that the vWbp-ClfA interaction is contact-time-dependent. The rupture forces averaged at 446 pN (*n* = 724 curves from 11 cells). As a control, we also probed ClfA^−^ cells with vWbp-modified tips (Fig. 4c). This resulted in appreciably decreased binding frequencies (3 ± 2%, *n* = 3 cells, Supplementary Table 4) compared to ClfA^+^ cells. The interaction of vWbp with ClfA thus appears to be stronger than the vWF-vWbp interaction, albeit much weaker than the vWF-vWbp-ClfA ternary interaction. This may indicate that in a pathophysiological context vWbp, produced and secreted by *S. aureus*, will stick to ClfA thereby facilitating bacterial binding to endothelial cell-exposed vWF. Taken together, the results also indicate that both the vWF-vWbp and ClfA-vWbp binary interactions are much weaker than the ternary complex. This implies that additional interactions must occur within the ternary complex to stabilize it.

### The vWF-A1 domain is insufficient for the ultra-strong vWF-vWbp-ClfA complex

The stability of the vWF-vWbp-ClfA ternary complex might be strengthened beyond that of the vWF-vWbp and vWbp-ClfA binary complexes if a direct vWF-ClfA interaction is established, owing to the structural aid of vWbp. One possibility is that ClfA directly interacts with domains adjacent to the vWF-A1 domain that vWbp binds to^18^. We therefore wondered if the vWF-A1 domain alone could form a ternary complex with vWbp and ClfA that is as strong as the one formed with full-length vWF.

**Fig. 5:**
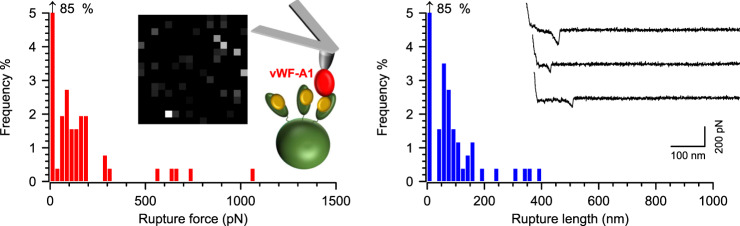
The vWF-A1 domain is insufficient for a highly stable vWF-vWbp-ClfA interaction. vWbp-treated ClfA^+^ *S. aureus* cells probed with vWF-A1-modified tips. Left panel: Histogram plot of rupture forces with insets showing the respective adhesion map (500 × 500 nm, 16 × 16 pixels, gray scale = 0–1.5 nN, each dot represents a binding event) and a cartoon of the experimental approach. Right panel: Histogram plot of the rupture lengths with insets showing three representative curves. Data shown come from one representative cell.

AFM tips were thus modified with recombinant vWF-A1 to probe vWbp-treated ClfA^+^ cells (Fig. 5). The resulting binding probability, rupture forces, and rupture lengths were 10 ± 4%, 188 ± 85 pN, and 81 ± 19 nm, respectively (*n* = 98 curves from 4 cells, Supplementary Table 5). The low forces observed for this interaction are within the range of the vWF-vWbp binary interaction (Fig. 4a). This finding strongly suggests that the vWF-vWbp bond would be the first to rupture in the ternary complex. More importantly, it implies that additional sequences in vWF are necessary for the high stability of the vWF-vWbp-ClfA complex, among which the A2, A3, and D′D3 domains adjacent to the A1 domain are potential candidates due to their likely close proximity to vWbp-bridged ClfA under tension, as mentioned above (see Fig. 1b).

### The ultrastability of the vWF-vWbp-ClfA complex requires the Fg γ-chain binding site of ClfA

**Fig. 6:**
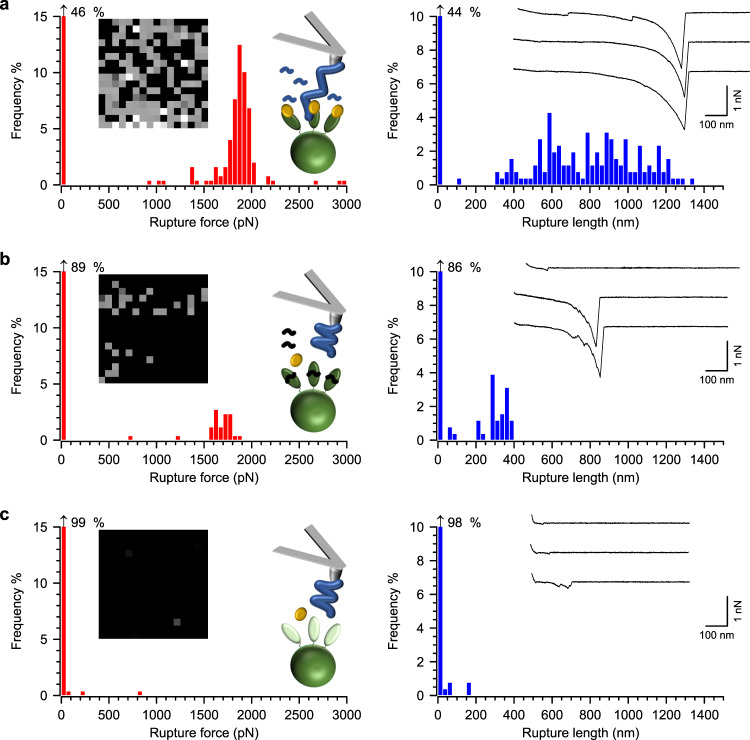
A dock, lock, and latch (DLL) interaction might be the key for a highly stable vWF-vWbp-ClfA complex. **a** ClfA^+^ *S. aureus* cells were first treated for 15 min with a peptide with a random sequence and then with recombinant vWbp before being probed with vWF-modified AFM tips. Data for a representative cell are shown. On the left are histograms of rupture forces with insets showing the respective adhesion maps (500 × 500 nm, 16 × 16 pixels, gray scale = 0–3 nN, each dot represents a binding event) and a cartoon in the top graph illustrates the experimental setup. The random peptide is illustrated by blue curled lines. On the right are shown histograms of the rupture lengths with insets showing three representative curves. **b** Data for ClfA^+^ *S. aureus* cells treated for 15 min with a peptide with the Fg γ-chain C-terminal sequence that binds to ClfA (black curled lines). vWbp was then added for 15 mins and the cells probed with vWF-modified tips. **c** ClfA_PY_ cells were treated with recombinant vWbp and then probed with vWF-modified AFM tips.

We therefore hypothesized that a direct ClfA-vWF DLL interaction might be involved in the formation of the ultrastable vWF-vWbp-ClfA complex. To test this, we first used a synthetic Fg γ-chain C-terminal peptide (residues 400–411), which would compete for ligand binding via the DLL mechanism^28^. As a control, we used a peptide with no sequence similarity to that of the Fg peptide and thus expected to be inactive if the DLL mechanism is involved in the interaction. ClfA^+^ cells were treated with each of the two peptides for 15 min prior to incubation with vWbp and probed with vWF-modified tips. Incubation with the control peptide led to rupture forces, rupture lengths, and binding frequencies (1830 ± 125 pN, 707 ± 92 nm, 36 ± 10%, *n* = 274 curves from three cells, Fig. 6a, Supplementary Table 6) in the same range as untreated cells (Fig. 2a, Supplementary Table 1). On the other hand, incubation with the synthetic Fg γ peptide led to a two-fold decrease in binding frequency of 19 ± 8% (*n* = 343 curves from 7 cells, Fig. 6b, Supplementary Table 6).

To complete our analysis, we used the *S. aureus* strain ClfA_PY_ that produces ClfA with amino acid substitutions P336S and Y338A; these two residues are essential for ultrastable ligand binding mediated by the DLL mechanism^28,35,37,38^. ClfA_PY_ cells incubated with vWbp before being probed with vWF-modified tips revealed a dramatic decrease in rupture forces, rupture lengths, and binding frequencies (109 ± 65 pN, 90 ± 64 nm, 3 ± 1%, respectively, *n* = 53 curves from 8 cells, Fig. 6c, Supplementary Table 6) compared to ClfA^+^ cells. These results indicate that formation of the vWF-vWbp-ClfA complex relies on a mechanism that shares important similarities with the ClfA-Fg γ-chain DLL interaction, thus supporting the idea that vWbp activates a direct, ultra-strong interaction between ClfA and vWF.

Interestingly, these results suggest that Fg may interfere in the ClfA-vWbp-vWF interaction. To test this hypothesis, we performed an experiment where full-length Fg was used as blocking agent. The results show, however, that addition of full-length Fg (at a maximum final concentration of 50 µg.ml^−1^) did not affect the magnitudes of forces or their frequencies in the vWF-vWbp-ClfA interaction (Supplementary Fig. 3 and Supplementary Table 7). Whole Fg thus does not appear to be a potent inhibitor of the vWF-vWbp-ClfA interaction. Available evidence suggests that the ClfA-vWbp-VWF and ClfA-Fg pathways are complementary in recruiting *S. aureus* to endothelial cells in whole blood^7^.

## Discussion

The ability of *S. aureus* to create metastatic infections as it spreads throughout the bloodstream is a major contributing factor to the poor prognosis of *S. aureus* bacteremia^39^. Underlying this ability are a set of surface adhesins that bind host proteins promoting strong adhesion to host tissue and cells^2^. vWF offers such an anchorage point for the bacteria on the blood vessel wall where it is either retained transiently on the surface of activated endothelial cells after its secretion or where circulating vWF binds subendothelial matrix collagen^12^. *S. aureus* needs both vWbp and ClfA to bind to vWF under shear flow^5–7^, yet the underlying molecular details of this interaction are not known.

We found that the ClfA-vWbp-vWF system is the most mechanostable ternary complex described so far, being able to resist ∼2000 pN forces. This contrasts with other known ternary interactions involving staphylococcal adhesins that rupture at weaker forces (~800 pN), such as the fibronectin (Fn)-binding protein A (FnBPA)-Fn-*α*_5_*β*_1_ integrin and the ClfA-Fg-*α*_V_*β*_3_ integrin ternary bridges^35,40^. These complexes rely on the high strength of the FnBPA-Fn and ClfA-Fg binary interactions^28,40^, which under mechanical tension unfold and reveal cryptic integrin-binding sites in the ligands (Fn or Fg), in turn allowing them to establish additional interactions with the integrins. This provides the complexes with an ability to outperform the classical strength of integrin binding (~100 pN) and increases their overall stability. In the case of the vWF-vWbp-ClfA ternary complex, both the vWbp-vWF and the vWbp-ClfA binary interactions are much weaker (~100 pN) than the ternary complex (2000 pN) suggesting that a different mechanism is at play.

What is the structural basis of the considerable strength of the vWF-vWbp-ClfA complex? The extreme strength of vWF-vWbp-ClfA is reminiscent of those reported for the mechanostable binary interactions involving a DLL mechanism, such as ClfA-Fg and SdrG-Fg^28,36^. The latter complex involves forces in the 2 nN range that are enhanced with the loading rate following the classical Bell–Evans theory^25^, very similar to the behavior of vWF-vWbp-ClfA. In the case of SdrG-Fg, the insertion of the Fg β-chain peptide in a trench between the N2 and N3 domains of SdrG triggers conformational changes at the C-terminus of the N3 domain that locks the peptide in place through a highly stable intricate hydrogen-bond network. Blocking the trench formed by the N2–N3 domain of ClfA with the Fg γ-chain C-terminal peptide or using a strain expressing mutated ClfA incapable of forming ultra-strong interactions, strongly suggest that ClfA binds vWF directly via a DLL mechanism. What if, alternatively, binding of vWF to vWbp has an allosteric effect increasing the stability of the vWbp-ClfA interaction via DLL? This hypothesis seems plausible, but that would also require that the vWF-vWbp bond is allosterically strengthened. This, on the other hand, is unlikely considering the protein sequences involved and their dissimilarity to MSCRAMM domains responsible for extraordinarily stable ligand binding^2,18,26^.

A truncated version of vWF that only contains the A1 domain was insufficient to support the high forces recorded for full-length vWF in complex with ClfA and vWbp. Therefore, it appears that additional sequences within vWF are required for a highly stable complex to form. Because extended vWF is a string of successive head-to-head and tail-to-tail disulfide-bridge bound protomers^41^, the most likely candidates are the A2, A3, and D′D3 domains that are adjacent to the A1 domain and that based on their sizes would be within approximate reach of vWbp-bridged ClfA (Fig. 1a). In particular, alignment of the N-terminal vWF-D′D3 domain with ClfA under load^2,42^ would favor a direct interaction. This idea is further substantiated by the fact that ClfA specifically binds to a free C-terminal peptide of the Fg γ-chain^27^ and related *S. epidermidis* SdrG to a free N-terminal peptide of the Fg β-chain^23^ via the DLL mechanism underlying the high mechanostability of these complexes^25,28,36^. Therefore, it is tempting to speculate that ClfA may directly bind a peptide sequence in the vWF-D′D3 N-terminus with the help of vWbp. Indeed, substrate promiscuity has been reported for the related MSCRAMM ClfB, which binds the Fg α-chain, cytokeratin 10, and dermokine via DLL^43^. How could vWbp catalyze binding of ClfA to vWF? First, as a bridge between the vWF-A1 domain and ClfA, vWbp may bring ClfA in close contact with the N-terminus of vWF^42^. Second, vWbp may directly activate a ClfA-vWF interaction through an allosteric effect on ClfA. Interestingly, such a function has indeed been described for staphylococcal coagulases, including vWbp^44^. vWbp acts as a cofactor by inserting its N-terminal peptide into an activation pocket of prethrombin, allosterically inducing its catalytic machinery^45^. The activated zymogen then binds and cleaves a sequence within the Fg β-chain that, interestingly, overlaps with the SdrG binding sequence^23^. It is an open question whether vWbp (or other staphylocoagulases) may in a similar way activate ClfA’s binding to its ligands.

In summary, our findings suggest a novel mechanism, not previously described, where the vWF-vWbp-ClfA ternary complex does not rely on the strength of a bridge provided by vWbp, as exemplified by the previously investigated FnBPA-Fn-α_5_β_1_ and ClfA-Fg-α_V_β_3_ complexes, but rather on the catalytic ability of vWbp that acts as a cofactor to trigger a very strong ClfA-vWF interaction. This notion is supported by several independent observations. First, both binary interactions are weak, while in Fn/Fg bridges, only one side is weak. Second, the 2 nN force and Bell–Evans behavior of the interaction perfectly match a DLL mechanism involving an MSCRAMM and a terminal peptide ligand^25,36^. Third, truncated vWF-A1 (vWbp-binding site) is not sufficient to establish high forces. Fourth, both structural and biochemical work, point to a strong binding of ClfA via DLL to either vWbp or vWF, the latter being supported by our control experiments described herein. Fifth, there is no crystallographic or biochemical data to support a model of strong binding between vWbp and vWF. For all of these reasons, a direct interaction between ClfA and vWF, catalyzed by vWbp, appears to be the most plausible explanation for the extreme stability of the complex. Future studies should aim to further decipher the underlying mechanism whereby vWbp catalyzes binding of ClfA to vWF as well as the exact sequences necessary for this ultrastable adhesin–ligand bond.

What are the implications of ultrastable adhesion via vWF-vWbp-ClfA in the pathophysiological context? It was previously shown that *S. aureus* adheres more to vWF under high than low fluid flow rates^5,6^. An explanation is offered by the fact that vWF extends under shear^14^, increasing the exposure of A1 domains and hence increasing the availability of binding sites for vWbp. Considering the size of a single *S. aureus* bacterium (~1 µm in diameter) and the spacing of A1 domains within a vWF oligomer^13^, it is conceivable that several vWbp molecules on the bacterial surface may bind to different A1 domains of a single vWF oligomer. Beyond this shear-dependent mechanism whereby bacterial adhesion to vWF may be maximized, we find that the vWbp-vWF bond is mechanically weak requiring the additional participation of ClfA to establish mechanically ultrastable bonds. Why bind so strongly to vWF? The disintegrin and metalloproteinase ADAMTS13, a plasma component, cleaves vWF in the A2 domain giving rise to shorter vWF fragments. It was found that the presence of ADAMTS13 activity diminished, but did not abolish, vWF-mediated adhesion of *S. aureus*^4,7,46^. It is, therefore, tempting to speculate whether ultrastable binding may offer *S. aureus* cells a means of adhering to shortened vWF fragments in the presence of ADAMTS13 when less abundant exposed A1 domains are available for interactions with multiple adhesins.

This study casts a new light on the importance of ultra-strong bonds formed by ClfA with blood circulatory proteins, highlighting the importance of this adhesin in *S. aureus* bloodstream infections. Our finding that vWbp potentiates extremely strong ClfA-mediated *S. aureus* adhesion to vWF is of biological relevance, as this represents a powerful means for the bacteria to strengthen their interaction with endovascular tissue under high shear stress. Importantly, it was recently reported that increased circulatory vWF contributes to the hypercoagulability precipitated by the severe acute respiratory syndrome coronavirus-2 (SARS-CoV-2)^47^ and could provide an explanation for co-infections with *S. aureus*^48^. The discovery that ClfA may bind vWF and Fg using the same binding site may be exploited for therapeutic interventions, using defined synthetic peptides.

## Methods

### Bacterial strains and growth conditions

Experiments were performed using the laboratory strain SH1000 *clf clfB fnbA fnbB* (*clfA*::Tn917, *clfB*::Tcr, *fnbA*::Tcr, *fnbB*::Emr) as control strain (ClfA^−^)^49^ or its derivatives that express ClfA_WT_ (ClfA^+^) or ClfA_P336S/Y338A_ (ClfA_PY_) from the multicopy shuttle plasmids pCF77^50^ and pCF77 PY^38^, respectively. Bacteria were routinely cultured on Trypto-Casein-Soy (T.C.S.) agar (Bio-Rad) or T.C.S. broth (Bio-Rad) supplemented with 10 µg·ml^−1^ chloramphenicol where necessary. For AFM experiments a single colony was transferred to 10 ml T.C.S. broth and the bacteria incubated with agitation at 37 °C until they reached stationary phase (~16 h).

### Recombinant vWbp and vWF-A1 domain production and purification

Recombinant vWbp was isolated as reported^51^. Recombinant vWF-A1 domain was produced in *E. coli* M15 cells harboring the plasmid pREP4 (Qiagen), originating from plasmid pQE30-vWF-A1 as previously reported^52,53^. The A1 domain contained an N-terminal His tag and was purified by Ni^2+^-affinity chromatography on a HiTrap chelating column (GE Healthcare, Buckinghamshire, UK).

### AFM tip and gold surface functionalization

Human plasma vWF (Sigma) was dissolved in PBS. This solution contained a mixture of vWF oligomers ranging in subunit length from *n* = 2 to *n* > 40, naturally occurring in human plasma. Gold-coated OMCL-TR4 AFM tips (Olympus) and gold-coated glass coverslips were immersed for 16 h in an ethanolic solution containing 16-mercaptododecahexanoic acid (0.1 mM) and 1-mercapto-1-undecanol (0.9 mM). Tips and coverslips were washed with ethanol, dried under a stream of N_2_, and immersed for 30 min in an aqueous solution of N-hydroxysuccinimide (NHS, 10 mg·mL^−1^) and 1-ethyl-3-(3-dimethylaminopropyl)-carbodiimide (EDC, 25 mg·mL^−1^). The resulting NHS-carboxyl ester exposing tips and surfaces were rinsed with ultrapure water and submerged in vWF (0.1 mg·mL^−1^) or vWbp (0.2 mg·mL^−1^) solutions for 1 h. Binding of the vWbp or vWF oligomers to the tips involved amide bond formation between the polypeptide backbones and the NHS-carboxyl esters. These are both covalent bonds that are even more stable than the ultrastable vWF-ClfA receptor–ligand interaction that we report here. Because of the random nature of the amide bond formation by which the proteins were bound to the carboxyl esters, it was very difficult to estimate the most probable number of subunits in the free-to-be-extended segments of vWF on the AFM tips. Considering the lengths of an extended vWF protomer and ClfA (vWbp extension cannot contribute to the length of the fully extended ternary complex at high force because bonds between it and one of the two other binding partners must have ruptured) as well as the range of rupture lengths that we observed (see the first “Results” section), we estimate that the vWF oligomer segments available for extension on our tips varied in protomer length from 1 to 5 units. Finally, tips and coverslips were gently rinsed with PBS and stored in the same buffer until they were used for experimentation. Prior to measurements, AFM tip spring constants were determined empirically by the thermal noise method^54^ and ranged between 0.05 and 0.10 N·m^−1^.

### AFM force spectroscopy experiments and parameters

Stationary phase cultures of bacteria were collected by centrifugation, washed twice with PBS, and resuspended in a volume of PBS equal to the original culture volume before being diluted 500× in PBS. This diluted bacterial cell suspension served as the experimental sample. For peptide blocking experiments, bacterial suspensions were incubated with peptide (final concentration of 0.2 mg·mL^−1^) for 15 min. A small volume of cellular suspension (peptide-treated or untreated) was then deposited on the surface of a polystyrene Petri dish and the cells allowed to adhere for 20 min. Then they were treated (or not) with vWbp (0.2 mg·mL^−1^) for 15 min. Finally, the cells were washed, the Petri dish filled with PBS and AFM experimentation performed. AFM was done using a JPK NanoWizard® 4 NanoScience AFM. Force-distance curves were collected in force mapping (force-volume) mode using a constant approach and retraction speed of 1 µm·s^−1^, a ramp length of 1–1.5 µm depending on the interaction, a contact force setpoint of 250 pN, no additional contact time unless specifically stated otherwise, and a closed z-loop. In experiments where cells were probed with protein-modified AFM tips, 32 × 32 or 16 × 16 pixel maps were recorded on a 500 × 500 nm area on top of the cells. In experiments where protein-modified surfaces were probed, 16 × 16 pixel maps were usually recorded on two 10 µm × 10 µm areas. In DFS experiments all the parameters described above were the same except for the retraction speed, which was varied over 2× intervals between 0.25 and 40 µm·s^−1^. ClfA and proteins exposed on AFM tips in this study were anchored to the cell wall or AFM tips, respectively, via covalent bonds that are stronger than the receptor–ligand interactions investigated here. Nevertheless, we vigilantly monitored for sudden or even progressive decreases in binding frequency during scanning, which can be taken as signs that anchorage points are ruptured. Here, we never observed such decreases in binding frequency.

### Force spectroscopy data analysis

For all force-displacement curves, the last rupture peak was fit using the extensible worm-like chain (eWLC) model of polymer extension, which better approximates polymer extension behavior in the high-force regime than the standard WLC model^55^. Rupture forces and lengths as well as *LR* were obtained along with eWLC fit parameters with the JPK data analysis software, which uses an algorithm to calculate these values from the fit data.

### Statistics and reproducibility

Statistical analyses were performed and graphs drawn with R. For DFS data, the Bell–Evans model was fit through the means of rupture force vs *LR* calculated for log-equispaced *LR* bins using nonlinear least-squares regression and the Port algorithm in R. Sample sizes and replicates are reported in the figure captions as well as in the Supplementary Information. Experiments were repeated at least twice. Differences in data distributions between samples were analyzed using two-way Student’s *T*-tests, two-way Mann–Whitney U tests, or ANOVA’s with Tukey post hoc tests for multiple comparisons and are reported in the Supplementary Tables. A *p*-value of <0.05 was considered significant.

### Reporting summary

Further information on research design is available in the Nature Research Reporting Summary linked to this article.

## Supplementary information

Supplementary Information

Description of Additional Supplementary Files

Supplementary Data 1

Reporting Summary

## Data Availability

The datasets generated or analyzed during the current study are available from the corresponding authors on reasonable request. Source data underlying plots shown in figures are provided in Supplementary Data 1.
