# Supplementary material for: Staphylococcus aureus vWF-binding protein triggers a strong interaction between clumping factor A and host vWF
Source: Commun Biol. 2021 Apr 12;4:453. doi: 10.1038/s42003-021-01986-6 (PMC8041789; doi:10.1038/s42003-021-01986-6)
Supplement: Supplementary file 1 — Supplementary Information [file 42003_2021_1986_MOESM1_ESM.pdf]

Staphylococcus aureus vWF-binding protein triggers a strong interaction between Clumping factor A and host vWF

Supplementary information

Albertus Viljoen[1,*], Felipe Viela[1], Marion Mathelié-Guinlet[1], Dominique Missiakas[2,3], Giampiero Pietrocola[4], Pietro Speziale[4,*] and Yves F. Dufrêne[1,5,*]

Supplementary Table 1 Mean rupture forces, rupture lengths and binding probabilities measured in single-molecule force spectroscopy experiments (SMFS) with vWF-modified tips and *S. aureus* $ClfA^+$ or $ClfA^-$ cells treated or not for 15 mins with vWbp.

Cell or parameter	*n*	F_{adh} (pN)[a]	L_{rupt} (nm)[b]	P_{adh} (%)[c]
$ClfA^+$ w/ vWbp				
1	125	1,794 ± 60	591 ± 169	49
2	195	1,788 ± 90	534 ± 120	76
3	119	1,752 ± 114	415 ± 134	46
4	41	1,412 ± 548	266 ± 118	16
5	139	1,752 ± 120	338 ± 75	54
6	94	1,727 ± 188	410 ± 121	37
7	125	2,173 ± 105	672 ± 156	49
8	46	2,240 ± 103	601 ± 280	18
9	65	2,502 ± 100	428 ± 80	25
10	70	2,424 ± 157	777 ± 342	27
11	127	2,650 ± 95	361 ± 78	50
12	81	2,105 ± 423	553 ± 176	32
13	540	1,713 ± 59	415 ± 91	53
14	254	1,921 ± 75	457 ± 95	25
**Mean**	**2,021**	**1,997 ± 359**	**487 ± 140**	**40 ± 17**[d,e]
$ClfA^+$ w/o vWbp				
1	3	908 ± 491	204 ± 129	1
2	8	1,943 ± 519	185 ± 57	3
3	12	1,540 ± 1229	231 ± 88	5
4	21	2,549 ± 179	375 ± 133	8
5	3	945 ± 1185	139 ± 74	1
6	2	1,681 ± 281	95 ± 11	1
7	19	2,319 ± 86	527 ± 90	7
8	25	268 ± 122	241 ± 92	10
9	23	397 ± 438	159 ± 84	2
**Mean**	**538**	**NA**	**NA**	**4 ± 3**[d]
$ClfA^-$ w/ vWbp				
1	4	852 ± 980	235 ± 63	2
2	3	198 ± 14	178 ± 12	1
3	2	1,969 ± 786	243 ± 57	1
4	11	2,042 ± 726	220 ± 41	4
5	1	0 ± 0	0 ± 0	0
6	15	2,650 ± 298	288 ± 52	6
7	1	0 ± 0	0 ± 0	0
8	3	997 ± 1,336	245 ± 163	1
9	1	0 ± 0	0 ± 0	0
10	3	142 ± 65	190 ± 76	1
**Mean**	**75**	**NA**	**NA**	**2 ± 2**[e]

[a]Adhesion force, mean ± standard deviation
[b]Rupture length, mean ± standard deviation
[c]Binding probability, mean ± standard deviation
[d]P_{adh} values of these samples are significantly different ($p < 0.001$, ANOVA with Tukey post-hoc test, two-way Student's T-test and two-way Mann-Whitney U test)
[e]P_{adh} values of these samples are significantly different ($p < 0.001$, ANOVA with Tukey post-hoc test, two-way Student's T-test and two-way Mann-Whitney U test)

Supplementary Table 2 Mean rupture forces, rupture lengths and binding probabilities measured for ClfA^{+} *S. aureus* cells probed with vWF-functionalized tips treated with vWbp for 15 mins.

**Cell or parameter**	***n***	F_{adh} **(pN)**[a]	L_{rupt} **(nm)**[b]	P_{adh} **(%)**[c]
1	24	1,961 ± 45	467 ± 98	9
2	142	1,979 ± 73	472 ± 87	55
3	72	1,944 ± 87	513 ± 225	28
4	145	1,804 ± 129	610 ± 199	57
5	50	1,852 ± 120	607 ± 176	20
6	37	1,793 ± 167	590 ± 47	14
**Mean**	**470**	**1,889 ± 83**	**543 ± 67**	**31 ± 21**

[a]Adhesion force, mean ± standard deviation
[b]Rupture length, mean ± standard deviation
[c]Binding probability, mean ± standard deviation

Supplementary Table 3 Mean rupture forces, rupture lengths and binding probabilities measured for vWbp-modified surfaces probed with vWF-modified AFM tips.

**Tip surface combination**	***n***	F_{adh} **(pN)**[a]	L_{rupt} **(nm)**[b]	P_{adh} **(%)**[c]
1	23	151 ± 78	66 ± 41	9
2	18	180 ± 79	61 ± 32	7
3	108	102 ± 37	110 ± 75	42
4	95	109 ± 39	101 ± 53	37
**Mean**	**244**	**136 ± 37**	**85 ± 25**	**24 ± 18**

[a]Adhesion force, mean ± standard deviation
[b]Rupture length, mean ± standard deviation
[c]Binding probability, mean ± standard deviation

Supplementary Table 4 Mean rupture forces, rupture lengths and binding probabilities measured for ClfA^{+} *S. aureus* cells probed with vWbp-functionalized tips.

Cell	*n*	F_{adh} (pN)[a]	L_{rupt} (nm)[b]	P_{adh} (%)[c]
ClfA^{+} S. aureus, contact time: 100 ms[d]				
1	7	163 ± 199	58 ± 32	3
2	8	924 ± 756	143 ± 90	3
3	11	86 ± 36	104 ± 52	4
**Mean**	**26**	**391 (86 -924)**[e]	**102 ± 43**	**3 ± 1**
ClfA^{+} S. aureus, contact time: 500 ms				
1	28	398 ± 450	86 ± 51	11
2	13	153 ± 70	116 ± 67	5
3	13	110 ± 78	128 ± 48	5
4	53	1,513 ± 542	292 ± 143	21
5	34	1,296 ± 749	151 ± 54	13
6	31	858 ± 662	93 ± 55	12
7	195	201 ± 108	162 ± 90	19
8	127	185 ± 116	193 ± 135	12
9	83	116 ± 62	66 ± 35	8
10	56	106 ± 88	92 ± 49	5
11	91	130 ± 85	60 ± 29	9
**Mean**	**724**	**446 (106 – 1513)**[e]	**131 ± 68**	**11 ± 5**[f]
ClfA^{-} S. aureus, contact time: 500 ms				
1	11	368 ± 529	58 ± 33	1
2	51	93 ± 47	83 ± 39	5
3	24	99 ± 50	91 ± 54	2
**Mean**	**86**	**187 (93 – 368)**[e]	**77 ± 17**	**3 ± 2**[f]

[a]Adhesion force, mean ± standard deviation
[b]Rupture length, mean ± standard deviation
[c]Binding probability, mean ± standard deviation
[d]minimal dwell time in absence of pause upon contact
[e]range shown in brackets.
[f]P_{adh} values of these samples are significantly different ($p < 0.05$, ANOVA with Tukey post-hoc test, two-way Student's T-test and two-way Mann-Whitney U test)

Supplementary Table 5 Mean rupture forces, rupture lengths and binding probabilities measured for $ClfA^+$ *S. aureus* cells probed with vWF-A1-modified tips.

Cell	*n*	F_{adh} (pN)[a]	L_{rupt} (nm)[b]	P_{adh} (%)[c]
1	39	135 ± 67	92 ± 47	15
2	21	248 ± 149	62 ± 26	8
3	21	272 ± 146	101 ± 64	8
4	17	98 ± 42	67 ± 17	7
**Mean**	**98**	**188 ± 85**	**81 ± 19**	**10 ± 4**

[a]Adhesion force, mean ± standard deviation
[b]Rupture length, mean ± standard deviation
[c]Binding probability, mean ± standard deviation

Supplementary Table 6 The dock, lock and latch mechanism of ClfA is necessary for an ultrastable ClfA-vWbp-vWF interaction. Mean rupture forces, rupture lengths and binding probabilities measured for SMFS experiments with *S. aureus* cells treated with vWbp and probed with vWF-modified tips. ClfA$^+$ *S. aureus* cells were treated with a control peptide or a Fg γ-chain peptide that blocks the ligand docking site in ClfA's DLL mechanism. ClfA$_{PY}$ *S. aureus* cells express mutated ClfA that cannot bind the Fg γ-chain.

Cell or parameter	*n*	F_{adh} (pN)[a]	L_{rupt} (nm)[b]	P_{adh} (%)[c]
ClfA$^+$ S. aureus treated with a random peptide and probed with vWF-modified tips				
1	141	1,960 ± 500	810 ± 248	55
2	57	1,710 ± 296	679 ± 181	22
3	76	1,820 ± 380	633 ± 159	30
**Mean**	**274**	**1,830 ± 125**	**707 ± 92**	**36 ± 10**[d,e,f]
ClfA$^+$ S. aureus treated with Fg γ-chain peptide and probed with vWF-modified tips				
1	116	2,091 ± 843	697 ± 158	45
2	33	1,558 ± 560	308 ± 91	13
3	1	1,629 ± 0	651 ± 0	0
4	29	1,649 ± 212	324 ± 44	11
5	144	1,708 ± 280	446 ± 111	56
6	8	1,148 ± 583	278 ± 185	3
7	12	94 ± 127	144 ± 99	5
**Mean**	**343**	**1411 ± 643**	**407 ± 203**	**19 ± 8**[d,e]
ClfA$_{PY}$ S. aureus probed with vWF-modified tips				
1	5	239 ± 344	94 ± 56	2
2	1	138 ± 0	15 ± 0	0
3	16	149 ± 235	215 ± 97	6
4	3	83 ± 50	111 ± 64	1
5	12	50 ± 19	42 ± 21	5
6	2	72 ± 19	91 ± 10	1
7	2	44 ± 8	31 ± 25	1
8	13	94 ± 46	119 ± 40	5
**Mean**	**54**	**109 ± 65**	**90 ± 64**	**3 ± 1**[d,f]

[a]Adhesion force, mean ± standard deviation
[b]Rupture length, mean ± standard deviation
[c]Binding probability, mean ± standard deviation
[d]mean ± standard error of the mean
[e]P_{adh} values of these samples are not significantly different (α = 0.05, ANOVA with Tukey post-hoc test and two-way Mann-Whitney U test)
[f]P_{adh} values of these samples are significantly different ($p < 0.05$, ANOVA with Tukey post-hoc test and two-way Mann-Whitney U test)

Supplementary Table 7 Mean rupture forces, rupture lengths and binding probabilities measured in single-molecule force spectroscopy experiments (SMFS) with vWF-modified tips and *S. aureus* $ClfA^+$ cells treated for 15 mins with vWbp following injection (or not) of Fg.

Cell or parameter	*n*	F_{adh} (pN)[a]	L_{rupt} (nm)[b]	P_{adh} (%)[c]
ClfA⁺ w/ vWbp before Fg injection				
1	64	1,970 ± 75	927 ± 132	25
2	26	2,042 ± 51	633 ± 142	10
3	66	1,905 ± 83	645 ± 235	26
**Mean**	**156**	**1,972 ± 69**	**735 ± 166**	**20 ± 9**[d]
ClfA⁺ w/ vWbp after Fg injection				
1	63	1,859 ± 90	325 ± 51	25
2	149	1,971 ± 71	354 ± 92	58
3	32	1,807 ± 146	325 ± 54	12
**Mean**	**244**	**1,879 ± 84**	**335 ± 17**	**32 ± 24**[d]

[a]Adhesion force, mean ± standard deviation
[b]Rupture length, mean ± standard deviation
[c]Binding probability, mean ± standard deviation
[d]P_{adh} values of these samples are not significantly different (α = 0.05, two-way Student's T-test and two-way Mann-Whitney U test)

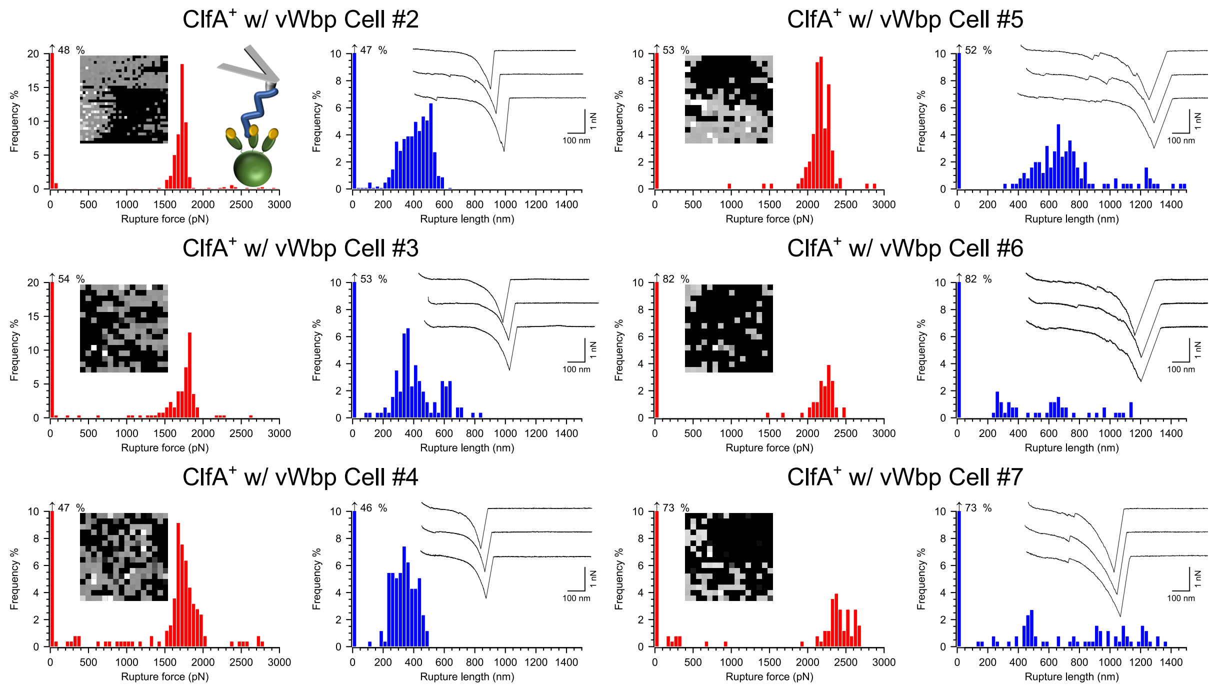

Supplementary Fig. 1 vWbp enhances ClfA-dependent binding of S. aureus to vWF. *S. aureus* cells expressing ClfA at high levels were treated with recombinant vWbp for 15 mins and then probed with vWF-modified AFM tips. Data for six representative cells is shown. For each cell histograms of rupture forces are shown on the left with insets showing the respective adhesion maps (500 nm × 500 nm, 32 × 32 or 16 × 16 pixels, each dot represents a binding event) and a cartoon in the top graph illustrates the experimental setup. Green ovals represent ClfA, golden spheres vWbp and blue lines vWF. On the right are shown histograms of the rupture lengths with insets showing three representative retraction profiles.

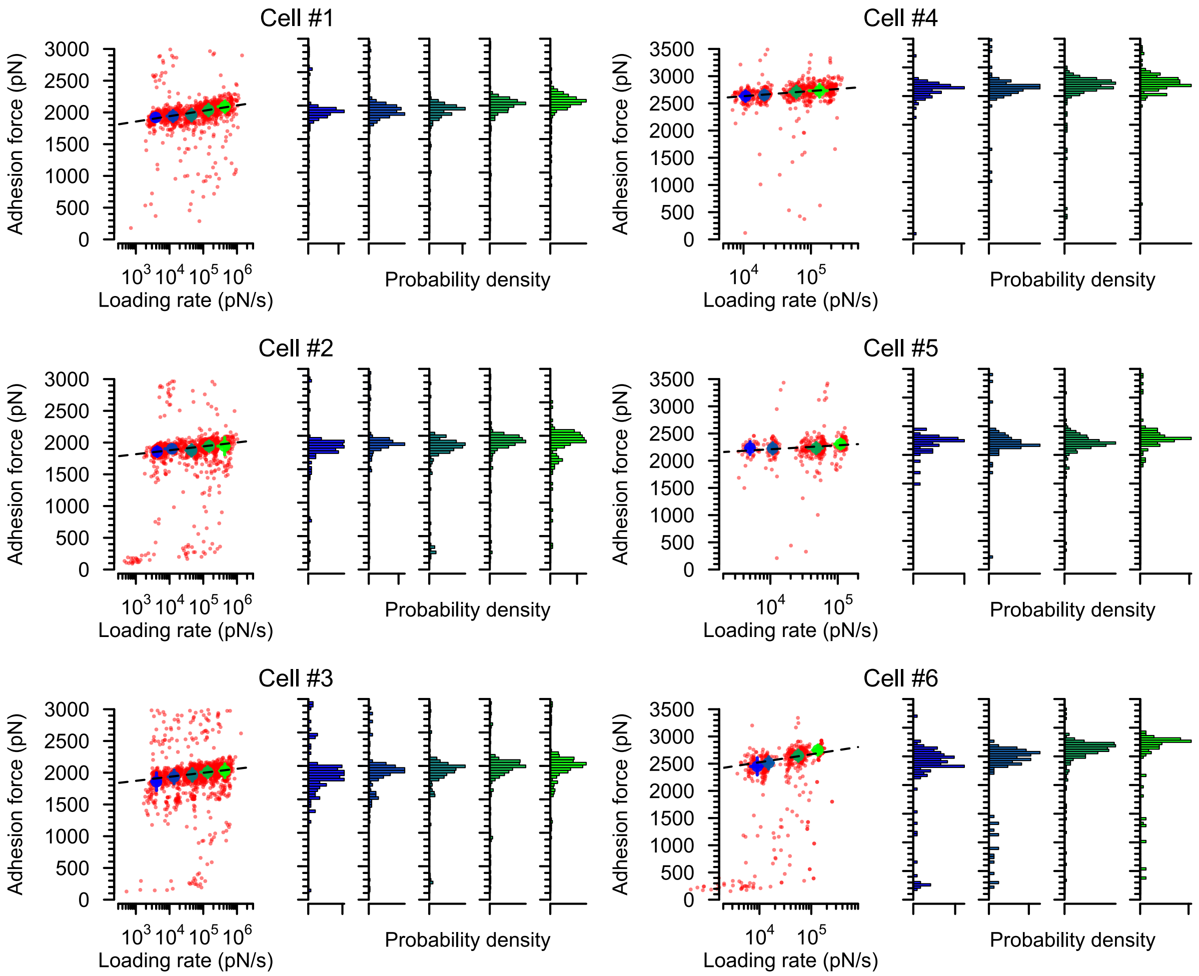

Supplementary Fig. 2 Force-loading rate dependence for the vWF-vWbp-ClfA ternary interaction follows Bell-Evans dynamics. Data of six representative cells is shown. On the left for each cell is shown the dynamic force spectroscopy plot of rupture force vs loading rate. The dotted line shows the extrapolated Bell-Evans fit through the most probable (mean) rupture forces and loading rates for five or four log-equispaced loading rate bins shown as solid circles. Error bars are the standard deviations. On the right are shown the corresponding histograms.

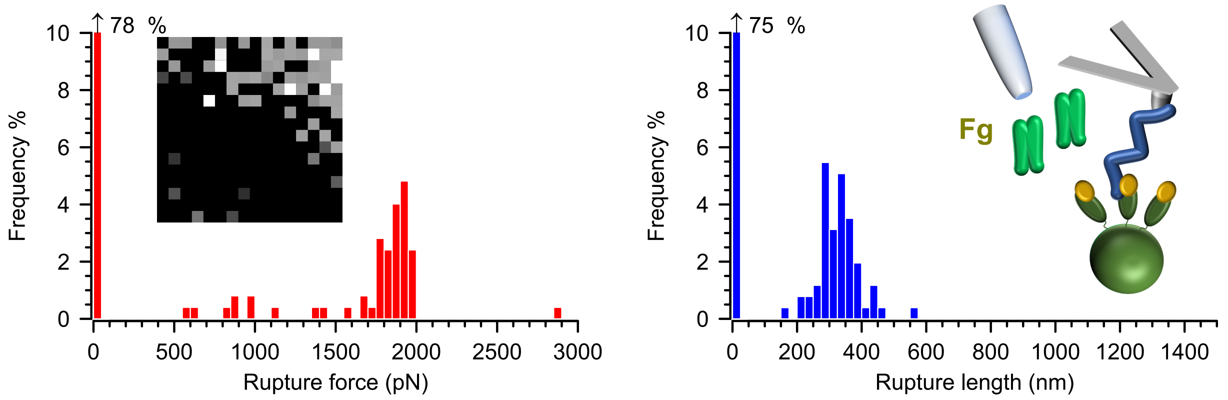

Supplementary Fig. 3 Fg has no effect on the vWF-vWbp-ClfA complex. ClfA$^+$ *S. aureus* cells were first treated with recombinant vWbp before Fg was injected into the Petri dish and the cells were probed with vWF-modified AFM tips. Data for a representative cell is shown. On the left are histograms of rupture forces with an inset showing the respective adhesion map (500 nm × 500 nm, 32 × 32 or 16 × 16 pixels, grey scale = 0 - 3 nN, each dot represents a binding event). On the right are shown histograms of the rupture lengths and a cartoon illustrates the experimental setup.
