# Supplementary material for: Staphylococcus aureus vWF-binding protein triggers a strong interaction between clumping factor A and host vWF
Source: Commun Biol. 2021 Apr 12;4:453. doi: 10.1038/s42003-021-01986-6 (PMC8041789; doi:10.1038/s42003-021-01986-6)
Supplement: Supplementary file 2 — Description of Additional Supplementary Files [file 42003_2021_1986_MOESM2_ESM.pdf]

Description of Additional Supplementary Files

File name: Supplementary Data 1

Description: Raw data used to draw each figure as tab-separated text (.tsv) files that can be imported into excel or opened with Notepad. In addition, we have included the R scripts used to draw the figures and tables and calculate statistics. These scripts can be run in RStudio Version 1.3.1073 or more recent.
